# Causality-Guided Machine Learning for Retinoblastoma Survival Prediction: Development and Comparative Evaluation Using SEER

**DOI:** 10.3390/medsci14030389

**Published:** 2026-07-14

**Authors:** Shijie Chen, Takashi Ishida

**Affiliations:** Department of Computer Science, School of Computing, Institute of Science Tokyo, Tokyo 152-8550, Japan; chen@cb.cs.titech.ac.jp

**Keywords:** retinoblastoma, surveillance, epidemiology, and end results (SEER), random survival forest, gradient boosting survival trees, DeepSurv, cox proportional hazards, survival prediction, causal inference, directed acyclic graph, nomogram

## Abstract

**Background**: Retinoblastoma (RB) is a rare pediatric malignancy characterized by small sample sizes and low event rates, where conventional association-driven feature selection may lead to unstable models, overadjustment, and limited generalizability. However, existing survival prediction studies lack a careful treatment of feature selection that accounts for underlying causal structure. **Objectives**: To develop and validate a causality-guided machine learning model for RB survival prediction by jointly incorporating survival time and survival status as outcome variables. **Methods**: We analyzed 1015 RB patients from the SEER database (1975–2020). A causality-informed feature selection framework was developed to address the challenges of rare-disease data. Specifically, candidate variables were evaluated through a three-step evidence-integration process: (1) univariate Cox proportional hazards (CPH) analysis for initial statistical screening; (2) causal structure learning using the PC algorithm on the variables retained from Step 1 to construct a directed acyclic graph (DAG) and exclude structurally inappropriate variables (colliders or descendants of the outcome); and (3) LASSO-based feature screening performed independently on the full set of candidate variables. The final features were obtained by taking the intersection of the variables retained from Step 2 and Step 3. Survival models were then trained using the selected features, with model comparison performed as a secondary step. **Results**: The proposed framework consistently identified four structurally and prognostically robust predictors—laterality, “SEER historic stage A”, “RX Summ”, and sequence number—through this evidence-integration process. Compared with conventional approaches, the causality-informed framework reduced the feature set while improving model stability and interpretability. Notably, compared with LASSO-only selection, which retained a larger set of variables, the causality-informed approach yielded a more parsimonious feature set with improved predictive performance, suggesting reduced overfitting in a low-event setting. Survival models trained on this refined feature set demonstrated reliable predictive performance, with the random survival forest achieving the highest discrimination (C-index = 0.739). Importantly, the selected predictors aligned with clinically plausible pathways in the learned DAG, supporting their causal relevance. **Conclusions**: This study demonstrates that incorporating causal structure into feature selection provides a more reliable and interpretable foundation for survival modeling in retinoblastoma. Rather than focusing on algorithmic comparison alone, our findings highlight that careful, causality-informed feature selection is critical for improving robustness in rare-disease prediction tasks. This framework may serve as a generalizable methodological template for other rare clinical settings prone to spurious associations.

## 1. Introduction

Retinoblastoma (RB) is an intraocular malignant embryonal tumor that originates from the retina and is the most common eye cancer in infants and young children. Approximately two-thirds of patients are diagnosed before the age of five, and the neonatal incidence rate is estimated to be 1 in 16,000 to 18,000 [1,2,3]. In China, RB is officially recognized as a rare disease because it is included in the First List of Rare Diseases issued by the National Health Commission [4]. In other countries and regions, the formal definition of rare disease may differ; for example, in the European Union, a rare disease is generally defined as one affecting fewer than 5 in 10,000 people [5], whereas in the United States it is commonly defined as one affecting fewer than 200,000 individuals [6]. Therefore, although the formal criteria vary across jurisdictions, RB is widely regarded as a rare pediatric malignancy. Compared with common diseases, rare diseases usually face challenges such as limited sample size, low event rates, and difficulty in external validation, which may increase the risk of model instability and overfitting [7]. In 20% to 30% of cases, both eyes are affected, while in 70% to 80% of cases, only one eye is affected [8]. Around the world, an estimated 5000 cases of retinoblastoma are diagnosed each year, with 250–300 cases in the United States and around 1000 cases in countries with large populations such as India and China [9,10]. The clinical manifestations of retinoblastoma include leukocoria, strabismus, eyelid swelling, pain, and glaucoma. The fundus may show single or multiple gray-white raised lesions, dilation of surface retinal blood vessels, and hemorrhage, which not only affect the eye and vision but can also be life-threatening [1,11]. Several factors influence the survival and prognosis of patients with RB, including disease duration and laterality. The survival and prognosis can differ significantly based on these factors [12,13]. The mortality rate also varies by region, with a rate of 5% to 11% in Europe and the United States, 30% in the Asia-Pacific region, and as high as 73% in African countries [14,15,16]. Therefore, it is essential to analyze these relevant factors and establish a survival prediction model to provide a basis for clinical treatment and timely intervention in high-risk groups.

Survival analysis is designed to model time-to-event data and is particularly suitable for clinical studies with censored observations, such as patients who have not experienced the event by the end of follow-up [17]. It considers both whether and when an event occurs, providing clinically meaningful outputs such as survival time, median survival, and survival probabilities [18]. Unlike binary classification models, survival analysis can incorporate censoring and the time dimension, and models such as the Cox proportional hazards (CPH) model provide interpretable measures such as hazard ratios [19]. Therefore, when time-to-event and censoring information is available, survival analysis is generally a more rigorous approach for survival prediction [20]. In contrast, classification models, such as logistic regression or XGBoost, usually predict survival status at a fixed time point, such as 5-year survival, and may ignore censoring and detailed survival time, leading to information loss and biased estimates [20,21]. Among survival methods, the CPH model remains a classic and widely used approach because of its interpretability, as it directly estimates hazard ratios and assesses variable importance through statistical tests [19,22,23]. However, its predictive accuracy can be limited. Recently, machine learning (ML)-based survival models, including random survival forest, gradient boosting, and deep learning, have improved predictive performance in various diseases, with concordance index (C-index) values often exceeding 0.7 in fields such as pulmonary and ophthalmic diseases [24,25,26,27,28]. ML-driven retinal imaging models have also been developed to predict systemic diseases such as diabetes [29]. Nevertheless, compared with the CPH model, ML-based survival models are often less interpretable because their decision processes are more difficult to explain clinically [30].

Despite these advancements, prognostic prediction research in RB remains limited. Recently, two related prediction studies have been reported. One study established a prognostic risk early-warning model based on XGBoost, which focused on 320 children with RB [31]. However, this model is a classification model that considers only 5-year survival status as the target variable. It was evaluated mainly using the Area Under the Curve (AUC) of the Receiver Operating Characteristic (ROC) curve, predicting survival at a fixed five-year time point. Thus, it cannot handle censored data or directly predict survival time. Additionally, the study excluded high-risk patients, such as those with an expected survival of less than six months, potentially introducing selection bias. Although the study compared XGBoost with Cox regression, it did not evaluate mainstream machine-learning survival methods within a time-to-event prediction framework [31]. Another recent study developed a random survival forest model based on the SEER database for patients with RB [32]. This represented an important advance because it adopted a survival modeling framework rather than fixed-time classification. However, it was limited to a single RSF framework and did not benchmark performance against other mainstream survival prediction methods [32]. In addition, existing studies remain limited in terms of validation strategy and have not incorporated causality-informed feature selection. Given the rarity of RB and the limited size of available datasets, further improving the robustness, interpretability, and generalizability of prediction models remains particularly important.

Feature selection represents another critical component in the development of robust models [33]. In survival modeling, commonly used feature selection strategies include stepwise selection procedures based on partial likelihood criteria and penalized regression methods such as the least absolute shrinkage and selection operator (LASSO), which are widely applied to address multicollinearity and high-dimensional covariate settings [34,35]; however, in complex observational clinical datasets, these methods may introduce unstable or biased predictors owing to confounding, collider bias, or inappropriate adjustment for mediating variables [36,37].

In recent years, integrating causal inference principles into predictive modeling pipelines has emerged as a promising strategy to improve model interpretability and transportability across populations, although this transportability benefit remains a hypothesis to be confirmed through external validation rather than a demonstrated property [38,39]. This motivation is driven by growing evidence that association-driven model development can yield misleading predictors and explanations in real-world clinical data. Models may attain strong internal performance by capturing correlations that are specific to the development setting and fail to transport, and retrospective feature construction can inadvertently introduce post-outcome information, leading to inflated estimates of predictive performance [40,41]. Moreover, conditioning on certain variables during modeling or cohort construction—particularly collider variables—can induce selection bias and create spurious associations that lack causal validity [42].

In this context, causal analysis is used not to estimate treatment effects, but to inform and constrain predictor selection by considering the underlying data-generating structure, typically represented using directed acyclic graphs (DAGs). By incorporating causal structure information, predictors that are structurally inappropriate or prone to bias can be identified and avoided, thereby enhancing model stability and interpretability at an early stage of model development [43,44].

In particular, causal inference can be incorporated into the feature selection stage, where predictors are selected based on their causal relevance to the outcome rather than purely statistical associations [38,39]. Such causality-informed feature selection aims to reduce spurious correlations and improve model robustness, and has been shown to either enhance predictive accuracy or maintain comparable performance while using a more parsimonious set of features. This approach has been increasingly explored in the ML literature and has demonstrated utility in various applications; however, its use in biomedical prediction remains limited and has not been systematically applied to RB, a rare pediatric malignancy characterized by small sample sizes and low event rates.

In addition, for ML-based survival models with relatively limited interpretability, introducing causal analysis may further improve the clinical interpretability of the modeling process by helping ensure that selected predictors are more consistent with clinically and biologically plausible pathways [30].

To address these gaps, this study aims to develop a survival prediction model for RB that jointly incorporates survival time (time to event) and survival status (event occurrence) as outcome variables, and represents one of the first attempts to integrate causality-informed feature selection into survival modeling for RB. A comprehensive model development framework is established, including training, validation, and test sets, together with a causality-informed feature selection strategy.

This strategy integrates conventional statistical evidence with causal structure exploration to identify prognostically relevant and structurally appropriate features before survival modeling, which is particularly important in rare diseases such as retinoblastoma where small sample size, low event rates, and limited clinical detail can amplify reliance on proxy signals and increase the risk of spurious feature selection [45].

The study then applies mainstream ML-based survival prediction methods, including random survival forests, gradient boosting survival trees, and DeepSurv. After benchmarking various approaches, the most suitable method for retinoblastoma data characteristics is selected to construct an optimal survival prediction model. This ensures the use of the most appropriate ML techniques while enhancing the model’s generalization performance.

Moreover, this study leverages the advantages of the SEER (surveillance, epidemiology, and end results) database [46], utilizing data from 1015 RB patients—significantly larger than previous studies [31,32]. Ultimately, this could effectively improve survival outcomes, prognosis, and the quality of life for affected children and their families.

## 2. Materials and Methods

### 2.1. Subjects

This study utilized data from the SEER database, which is one of the largest publicly available cancer registries in the United States, covering approximately 28% of the U.S. population [47]. Given the rarity of retinoblastoma, the SEER database provides a valuable resource for assembling a sufficient sample size. The SEER data used for this study were first accessed on 17 May 2025. This retrospective analysis extracted data on all retinoblastoma patients recorded in SEER, including demographics, survival outcomes, cause of death, socioeconomic status, geographic information, diagnostic details, eye laterality, treatment, and tumor characteristics. All data obtained from the SEER database were fully de-identified, and the authors did not have access to any identifying information about the participants during or after the study. Due to the limitations of the publicly available SEER dataset, data collected between 1975 and 2020 were used in this study. After excluding patients and variables with more than 50% missing values, a final cohort of 1015 patients and 19 variables were retained for analysis (originally 1020 patients and 50 variables).

### 2.2. Modeling

**Figure 1:**
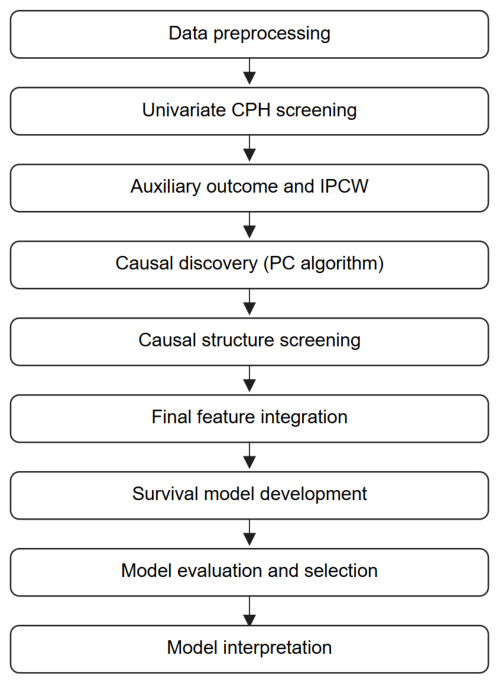
Workflow of the causality-informed survival prediction modeling process.

To develop a highly accurate and robust survival prediction model, we adopted a multi-step modeling framework that integrates traditional statistical methods, ML-based techniques, and causality-informed variable selection to guide model construction. The overall modeling workflow is shown in Figure 1. The model development process is as follows.

#### 2.2.1. Baseline Assessment and Data Preprocessing

To ensure interpretability and identify significant predictors, we conducted a CPH univariate analysis. This served as part of the baseline assessment and provided an overview of the data distribution and its relationship with survival outcomes.

Unlike previous RB prediction studies [31,32], which relied on a single training-validation split, our study implemented a more rigorous strategy by randomly dividing the dataset into training, validation, and test sets (8:1:1), thereby enhancing model robustness and evaluation reliability.

Given the presence of missing values in the dataset, we employed the Multiple Imputation by Chained Equations (MICE) [48,49] method, which is a flexible and practical approach for handling missing data in medical research. To avoid information leakage, the imputation model was fitted on the training set and subsequently applied to the validation and test sets during model development.

#### 2.2.2. Causal Structure Exploration (Auxiliary Outcome Construction for Causal Discovery)

To enable causal structure exploration using constraint-based methods, an auxiliary outcome construction was performed exclusively for causal discovery purposes. Because standard constraint-based causal discovery algorithms are not designed to directly accommodate right-censored time-to-event outcomes, a transformation of the survival outcome was required solely at this stage.

Specifically, a binary outcome was constructed to indicate whether the event of interest occurred within a predefined time horizon of 60 months, corresponding to a clinically meaningful medium-term survival endpoint.

To account for right censoring and mitigate selection bias arising from incomplete follow-up, inverse probability of censoring weighting (IPCW) [50] was applied. The censoring distribution was estimated using the Kaplan–Meier estimator, and each individual was assigned a weight equal to the inverse of the estimated probability of remaining uncensored at their observed follow-up time.

Based on these weights, a weighted resampling procedure was conducted to generate a pseudo-population in which the auxiliary binary outcome could be treated as approximately independent and identically distributed. This IPCW-adjusted dataset was used exclusively as input for causal structure learning.

Causal structure exploration was then performed using the PC (Peter–Clark) algorithm [51], a constraint-based causal discovery method. The PC algorithm infers conditional independence relationships from observational data and constructs a DAG, representing plausible causal structures among variables. Detailed PC-algorithm settings are provided in Supplementary Table S3.

Based on the learned causal DAG, variables were further categorized according to their structural proximity to the survival outcome. Specifically, variables were classified into three tiers: (i) Tier 1 variables, which exhibited direct structural connections to the survival outcome; (ii) Tier 2 variables, which were linked to the outcome through a single indirect causal path and typically acted as mediators; and (iii) Tier 3 variables, which were connected to the outcome only through two or more indirect paths and were considered structurally distal.

Variables that were structurally unrelated to survival outcomes or identified as potential sources of bias—such as outcome descendants or collider variables—were excluded from the causality-informed screening branch. In accordance with the predefined causal screening strategy, only Tier 1 and Tier 2 variables were retained as causally plausible predictors for final feature integration, while Tier 3 variables were excluded to reduce structural noise and enhance model stability.

Importantly, this auxiliary outcome construction and IPCW-adjusted resampling were applied solely to facilitate causal structure learning and did not alter the definition of outcomes used in any subsequent survival modeling analyses, all of which were based on the original time-to-event outcome and censoring information.

#### 2.2.3. Model Construction

Following data preprocessing, we constructed survival prediction models using both traditional statistical and ML-based methods. All models were implemented in Python (version 3.10.16).

(1) Cox Proportional Hazards Model [22]

The CPH model was implemented as a traditional method for survival prediction. Feature selection was performed using a three-step evidence-integration strategy combining univariate analysis, causal screening, and Lasso regularization [52,53] to identify the most relevant predictors and improve model performance. First, univariate CPH analysis was used for initial statistical screening. Second, PC algorithm-based causal screening was performed on the variables retained from the univariate analysis to identify variables with plausible structural relevance to the survival outcome. Third, Lasso-based feature screening was performed independently on the full set of candidate variables. The final feature set was obtained by taking the intersection of variables retained after causal screening and variables selected by Lasso regularization. The optimal regularization parameter (alpha) for Lasso was determined using 5-fold cross-validation.

(2) ML Methods

ML-based survival prediction methods, including RSF [54], GBST [55], and DeepSurv [56,57], were employed, chosen for their ability to capture complex, non-linear relationships in the data [58,59]. All ML-based models were trained using the same set of variables as the CPH model.

Hyperparameter optimization for each ML-based model was conducted using grid search, which systematically evaluated combinations of hyperparameters to identify the configuration with the best predictive performance based on the C-index. The hyperparameter search spaces explored during model development are detailed in Supplementary Table S1.

#### 2.2.4. Model Selection

To assess the strengths and limitations of each method, we systematically evaluated the performance of all constructed models. This comparative analysis aimed to determine the most accurate and robust predictive approach. Model performance was quantified using the C-index, a standard metric for evaluating the discriminative ability of survival models [60]. The model achieving the highest C-index on the test set was selected as the final predictive model for this study.

#### 2.2.5. Model Interpretability

ML-based methods cannot directly output the contribution of individual features to the prediction. Thus, to obtain the interpretability of the final model, we applied the Local Interpretable Model-agnostic Explanations (LIME) method [61]. LIME learns a locally interpretable model around each individual prediction and explains its output for any classifier by identifying which features contributed most to the prediction for that specific instance [61].

The causal DAG derived from the PC algorithm was further used to contextualize model explanations and facilitate interpretation of the clinical relevance and structural role of selected variables.

Together, these approaches enable a clearer understanding of the model’s decision-making process, even when the underlying model is complex and non-linear.

### 2.3. Data Analysis

Quantitative data were described as mean ± standard deviation for normally distributed variables, and as median (Q1, Q3) for non-normally distributed variables. Qualitative data were expressed as percentages.

Univariate analysis was conducted to assess the association between specific factors and survival outcomes. The CPH model was employed to analyze the variables influencing survival.

All statistical analyses were conducted using Python (version 3.10.16) and R (version 4.2.2). R was used for the construction of nomograms and for reproducing previously published methods for comparative analysis.

### 2.4. Evaluation Metrics

The predictive performance of the model was finally evaluated using the C-index, which quantifies the model’s ability to correctly rank survival times, the AUC of the ROC curve for time-specific classification performance, and the Brier scores, which assess the accuracy of probabilistic predictions by capturing both calibration and discrimination aspects over time.

Univariate analysis was conducted using the CPH model, with two-tailed Wald tests applied to assess statistical significance (*p* < 0.05).

This combination of performance metrics and statistical evaluation ensured a comprehensive and rigorous assessment of the model development process.

## 3. Results

### 3.1. Patient Characteristics and MICE Imputation

In this study, 1015 subjects meeting the inclusion and exclusion criteria were included, consisting of 500 males (49.26%) and 515 females (50.74%) with an average age of 1.45 ± 3.41 years. The full cohort included 87 patients who experienced the event of interest. The dataset was first randomly divided into training (812 patients), validation (101 patients), and test (102 patients) sets in an 8:1:1 ratio. Missing values were subsequently handled using the MICE method according to the preprocessing procedure described in Section 2. The numbers of events in the training, validation, and test sets were 67, 8, and 12, respectively.

Baseline demographic and clinical characteristics are summarized in Table 1.

### 3.2. Univariate Survival Analysis and Baseline Feature Screening

Univariate CPH analysis was first performed to evaluate the marginal associations between candidate variables and survival outcomes. Six features were identified as statistically significant (*p* < 0.05):

■ X1: gender
■ X2: laterality
■ X3: SEER historic stage A
■ X4: sequence number
■ X5: first malignant primary indicator
■ X6: RX Summ–Surg Oth Reg/Dis

Variable definitions are provided in Table 1.

These results provided an initial statistical screening of potential prognostic factors and served as an empirical starting point for subsequent structure-aware refinement.

### 3.3. Causal Structure Exploration and DAG-Based Feature Refinement

To further contextualize and structurally validate the candidate features identified in univariate analysis, causal structure exploration was performed using the PC algorithm.

**Figure 2:**
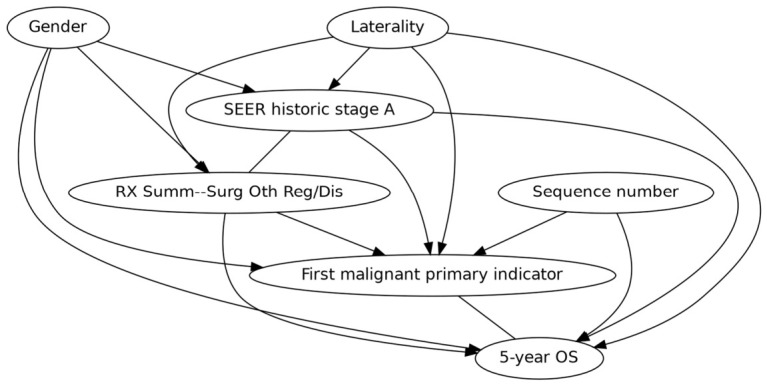
DAG: Y as Outcome Variable—Causal Relationship Network.

Based on conditional independence relationships inferred from the training data, a DAG describing the plausible causal structure among the retained variables and the constructed survival outcome was obtained (Figure 2).

The learned DAG revealed that most candidate features exhibited stable structural connections to the outcome. However, two variable pairs—(X3–X6) and (X5–Y)—were connected by undirected edges, suggesting that their causal directions could not be uniquely identified from observational data alone.

Specifically:

- The relationship between X3 and X6 admitted three possible configurations: X3 → X6, X6 → X3, or X3 ⊥ X6.
- The relationship between X5 and the outcome (Y) similarly admitted three possibilities: X5 → Y, X5 ⊥ Y (Y → X5 is impossible because the survival outcome occurs after and cannot causally influence prior clinical features).

**Figure 3:**
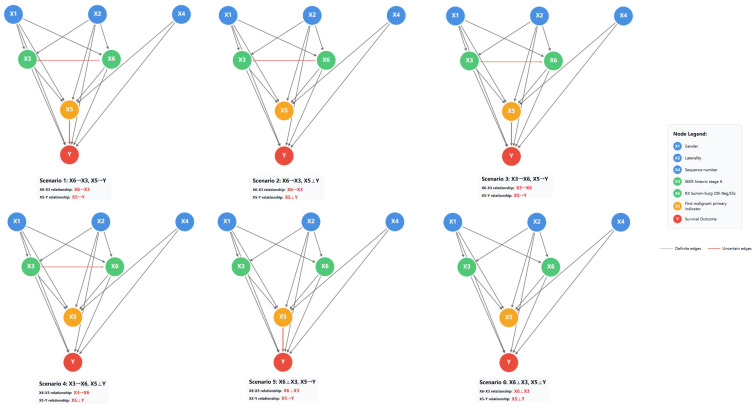
DAG Possibilities with Uncertain Edges—6 Valid Scenarios.

These ambiguities resulted in six theoretically plausible DAG configurations consistent with the conditional independence structure inferred by the PC algorithm (Figure 3). In practical terms, these alternative configurations mainly clarified which uncertain edge was relevant to feature retention: the X3–X6 orientation did not affect downstream eligibility, whereas the X5–Y orientation was structurally decisive for excluding X5.

Across all six plausible DAG configurations, the causal direction between X3 and X6 did not affect variable eligibility, as both variables maintained direct or near-direct structural paths to the outcome and were therefore consistently classified as tier 1 or tier 2 predictors.

In contrast, the causal orientation between X5 and Y was structurally decisive:

- When X5 ⊥ Y, X5 lacks causal relevance to the outcome and was therefore excluded from downstream modeling.
- When X5 → Y, although X5 appears as a direct upstream node of the outcome, it simultaneously aggregates information from multiple antecedent variables (including X1, X2, X3, X4, and X6). In this setting, X5 functions as a structural summary variable rather than an independent prognostic factor. Although such a summary variable could potentially improve discrimination in a purely predictive model, retaining it would reduce the structural interpretability of the selected feature set and may introduce redundancy or overadjustment. To avoid redundancy, prevent overadjustment, and preserve the interpretability of upstream causal pathways, X5 was excluded from the final predictive feature set across all DAG-consistent scenarios. This strategy ensured that retained predictors represented distinct and interpretable contributors to survival risk rather than composite downstream manifestations.

Accordingly, gender (X1), laterality (X2), SEER historic stage A (X3), sequence number (X4), and RX Summ (X6) were retained for downstream survival modeling. This causality-guided strategy balances causal plausibility with transparency regarding residual structural uncertainty.

To assess whether the learned causal structure was sensitive to the fixed-time endpoint definition, we further repeated the causal discovery procedure using an IPCW-adjusted 36-month binary survival endpoint. The resulting DAG was consistent with the primary 60-month DAG, with the same major predictors remaining structurally connected to the survival-related auxiliary outcome. The interpretation of the first malignant primary indicator as a structurally downstream summary variable was also unchanged. These findings suggest that the main feature-structural relationships were not driven solely by the specific 60-month cutoff and support the robustness of applying DAG-informed feature screening to downstream time-to-event survival modeling. The 36-month sensitivity DAG is provided in Supplementary Figure S2.

### 3.4. Construction of Cox Proportional Hazards Model

**Figure 4:**
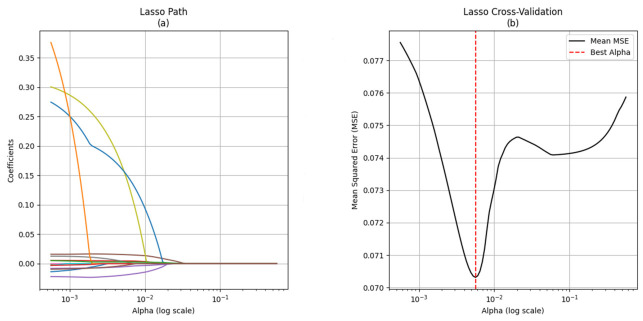
LASSO feature-screening analysis: (**a**) coefficient paths across alpha values, where each colored line represents the coefficient trajectory of one candidate variable; (**b**) five-fold cross-validation curve showing the mean squared error across alpha values, with the dashed red line indicating the selected optimal alpha value.

In parallel with the causality-informed screening process, LASSO-based feature screening was performed independently on the full set of candidate variables to further control model complexity and mitigate multicollinearity, as shown in Figure 4a,b. Five-fold cross-validation determined the optimal alpha value for Lasso to be 0.00525. The selection process identified nine variables: year of diagnosis, laterality, “diagnostic confirmation”, “SEER historic stage A”, “RX Summ”, “sequence number”, age, median household income, and “Rural-urban continuum code” (definitions in Table 1). The resulting CPH model yielded a C-index of 0.652 on the test set.

By integrating the variables retained after causal screening with those selected by LASSO regularization, four overlapping variables, laterality, “SEER historic stage A”, “RX Summ”, and “sequence number”, were retained as the final predictive feature set. A refined CPH model was constructed using these variables, which resulted in an improved C-index of 0.687 on the test set. The final CPH model, derived from the training set, is presented in Table 2. It should be noted that several RX Summ categories showed very wide confidence intervals, and one category yielded a large hazard ratio. This pattern may reflect sparse-cell instability caused by the low event rate and small subgroup counts.

### 3.5. Construction of ML-Based Model

Based on consistency across univariate Cox analysis, causal structure screening, and LASSO-based feature selection, four variables—laterality, “SEER historic stage A”, “RX Summ”, and “sequence number”—were retained as the final predictive feature set for downstream survival modeling as well.

Using this causality- and statistics-informed feature subset, ML-based survival models, including RSF, DeepSurv, and GBST, were constructed.

To achieve optimal model performance, hyperparameter tuning was performed using grid search within the training set, and the optimized configurations for each ML-based model are summarized in Table 3.

On the independent test set, the resulting C-index values were 0.739 for RSF, 0.670 for DeepSurv, and 0.668 for GBST, respectively. In comparison, the refined multivariable CPH model achieved a lower C-index of 0.687.

These results indicate that, when trained on a feature set jointly supported by univariate statistical significance, causal plausibility, and multivariable stability, ML-based survival models—particularly RSF—demonstrated improved discriminative performance compared with the classic CPH model, although the magnitude of improvement was moderate.

### 3.6. ML Method (RSF) vs. Classic Method (CPH)

As the model with the highest discriminative performance in terms of the C-index, the RSF achieved a C-index of 0.739, outperforming the CPH model, which yielded a C-index of 0.687. To further compare the predictive performance of the two models, we evaluated their AUC metrics.

As shown in Supplementary Figure S1, the ROC curve based on 5-year overall survival (OS) demonstrated that RSF achieved an AUC of 0.713, which was higher than that of the CPH model (AUC = 0.683), indicating an advantage of RSF in long-term survival discrimination.

**Figure 5:**
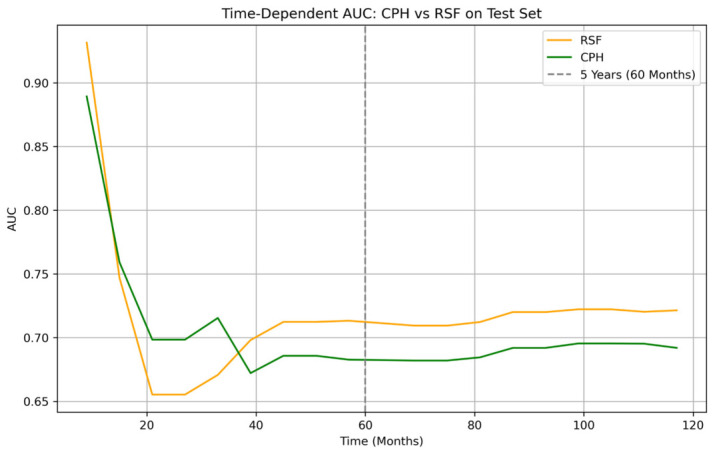
Time-Dependent AUC: CPH vs. RSF on Test set.

Time-dependent AUC analysis (Figure 5) revealed dynamic differences in model performance across follow-up periods. During the early follow-up stage (1 year), RSF exhibited superior discriminative ability compared with CPH (RSF: 0.946 vs. CPH: 0.887). However, during the intermediate follow-up interval (14 to 35 months), CPH showed slightly higher AUC values (e.g., at 14 months: RSF 0.743 vs. CPH 0.759; at 35 months: RSF 0.668 vs. CPH 0.715). From 36 months (3 years) onward, RSF again outperformed CPH (3 years: RSF 0.658 vs. CPH 0.646) and consistently maintained higher AUC values throughout the later follow-up period.

Overall, although RSF demonstrated superior performance in predicting 1-, 3-, and 5-year survival, its advantage over CPH was not uniform across all time intervals. The CPH model retained competitive and stable predictive performance during the intermediate follow-up period. These findings suggest that CPH and RSF capture complementary aspects of survival risk, and that integrating the proportional hazards structure of CPH with the nonlinear modeling capacity of RSF may provide a more balanced and clinically meaningful approach to survival prediction.

## 4. Discussion

In this study, we developed and evaluated a causality-informed survival prediction framework for RB using the SEER dataset. The variable selection framework integrated three complementary sources of evidence: univariate CPH analysis as an initial statistical filter, PC algorithm-based causal structure exploration to evaluate structural plausibility, and LASSO-based multivariable regularization to further control model complexity and multicollinearity. Only variables that demonstrated consistent support through this evidence-integration process were retained in the final predictive feature set. Compared with traditional CPH modeling, the ML-based survival models showed stronger predictive performance, and the final selected model achieved the best overall discrimination among the evaluated approaches. These four variables—laterality, “SEER historic stage A”, “RX Summ”, and “sequence number”—were selected through this integrated feature-selection framework and formed the basis for both the refined CPH model and the ML-based survival models.

**Figure 6:**
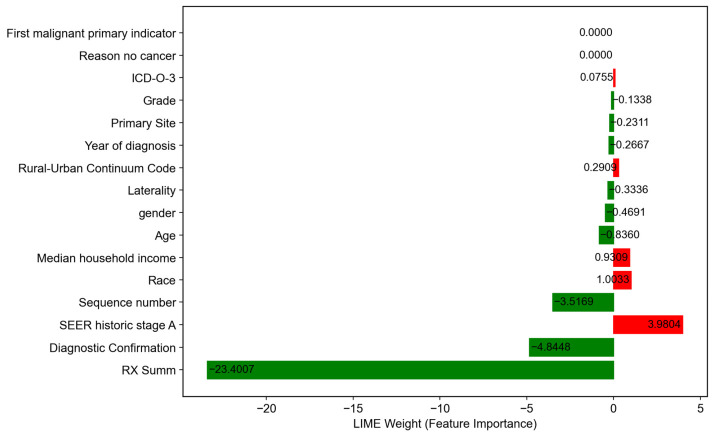
LIME of RSF Model.

To further assist the interpretation of the final RSF model, we additionally applied the LIME method as a supplementary post hoc explanation tool. In this study, LIME was not used to define the final set of prognostic factors, but rather to examine whether the local explanation patterns of the RSF model were broadly consistent with the predictors retained in the final analysis and with the directions of association suggested by the CPH model. In both models, “SEER historic stage A”, “RX Summ”, and “sequence number” emerged as the primary predictors of RB prognosis. Since RSF, as a non-linear ensemble method, does not directly provide interpretable coefficients or hazard ratios, LIME was applied as a post hoc explanation tool to examine the relative contribution of individual features to the model’s predictions. Among the features ranked by LIME, the top four in terms of absolute importance were “RX Summ” (−23.401), “Diagnostic Confirmation” (−4.845), “SEER historic stage A” (+3.980), and “sequence number” (−3.517) (Figure 6). Notably, three of these four features—“RX Summ”, “SEER historic stage A”, and “sequence number”—all belonged to the four variables retained through our integrated feature-selection framework, providing indirect evidence that the features identified by our framework are indeed clinically and prognostically meaningful. “Diagnostic Confirmation”, which ranked second in LIME importance, was not retained in the final model: it was excluded as it did not reach statistical significance in univariate CPH analysis (*p* ≥ 0.05) and therefore did not enter subsequent causal refinement. This pattern illustrates a key limitation of purely data-driven importance metrics: a variable may exert strong local predictive influence within a trained ensemble model while lacking the statistical association and causal interpretability required for reliable prognostic inference. Although LIME operates as a local linear approximation and its feature weights should not be interpreted as equivalent to CPH hazard ratios, the convergence between the LIME-derived importance ranking and our final selected feature set lends additional support to the robustness of our variable selection strategy.

A distinctive methodological contribution of this study is the integration of a causality-informed variable selection strategy into the survival modeling framework. The selection procedure followed a three-step evidence-integration framework: univariate CPH analysis first identified six statistically significant candidate variables (X1–X6); PC algorithm-based causal structure exploration then evaluated the structural plausibility of each candidate; and LASSO-based feature screening was performed independently on the full set of candidate variables. The final feature set was defined as the intersection of variables retained after causal screening and variables selected by LASSO regularization. This strategy offers important advantages over conventional single-criterion selection. The PC algorithm was applied to the training data to construct a DAG describing the plausible structural relationships among candidate variables and the survival outcome (Figure 2). The learned DAG revealed two structurally ambiguous variable pairs: the relationship between X3 (SEER historic stage A) and X6 (RX Summ) admitted three causal configurations (X3 → X6, X6 → X3, or X3 ⊥ X6), and the relationship between X5 (First malignant primary indicator) and the outcome Y similarly admitted three possibilities (X5 → Y, X5 ⊥ Y, or Y → X5—the last being structurally impossible as clinical features cannot be caused by future survival outcomes). These ambiguities yielded six theoretically plausible DAG configurations (Figure 3). The directional uncertainty between X3 and X6 did not affect variable eligibility, as both variables maintained direct or near-direct structural paths to the outcome across all six scenarios and were therefore consistently classified as Tier 1 or Tier 2 predictors. In contrast, the causal orientation of X5 was structurally decisive: when X5 ⊥ Y, X5 lacked causal relevance and was excluded; when X5 → Y, X5 simultaneously aggregated information from multiple antecedent variables including X1, X2, X3, X4, and X6, functioning as a structural summary variable rather than an independent prognostic factor. Retaining X5 in this scenario would risk overadjustment bias and obscure the interpretable causal contributions of upstream predictors [37]. Accordingly, X5 was excluded from all DAG-consistent scenarios. Gender (X1), despite passing both univariate statistical screening and causal structure evaluation, was not retained by LASSO regularization, suggesting that its apparent marginal association with survival is largely explained by or shared with the remaining predictors in the multivariable setting. This evidence-integration strategy ensured that retained predictors represented distinct, interpretable, and causally plausible contributors to survival risk.

“SEER historic stage A” and laterality were retained as two of the four final predictors. Given their structurally linked positions in the learned DAG, their prognostic interpretation is best understood jointly. “SEER historic stage A” is derived from the Collaborative Stage (CS) for cases diagnosed between 2004 and 2015 and the Extent of Disease (EOD) for cases from 1973 to 2003, representing a clinically meaningful simplified staging classification [46]. In the learned DAG (Figure 2), laterality appeared as a root node—receiving no incoming edges from any other variable—while “SEER historic stage A” occupied a central downstream position, receiving a directed edge from laterality and contributing directly to the survival outcome as a Tier 1 predictor. This DAG structure encodes a specific causal hypothesis: laterality influences survival primarily by shaping the distribution of downstream tumor stage, which in turn drives mortality risk, rather than acting as an independent direct determinant of outcome. The clinical basis for this pathway is well established: bilateral retinoblastoma is almost exclusively associated with germline RB1 mutations and tends to be detected earlier due to involvement of both eyes and proactive family screening, whereas unilateral cases more often present at advanced stages. Mohammad et al. reported that 88% of unilateral RB cases presented with advanced-stage disease (IIRC group D/E) compared to only 46% of bilateral cases, yet found no significant difference in metastasis, secondary neoplasms, or mortality between the two laterality groups (mortality *p* = 0.8193). This dissociation—marked staging differences alongside equivalent mortality outcomes—is precisely what a mediation model predicts: laterality influences the staging profile and downstream treatment decisions, but once these mediating factors are accounted for, the direct mortality difference between laterality groups disappears [62]. In our final model, “SEER historic stage A” remained an important prognostic factor. The results from the CPH model (Table 2) confirmed that localized tumors exhibited a significant protective effect relative to distant disease. This finding aligns with previous studies [63,64], which have demonstrated that tumor growth and distant metastasis are key contributors to mortality in children with retinoblastoma [65]. For laterality, subgroup-level hazard ratios did not reach statistical significance in the multivariable CPH model (Table 2). However, this should not be interpreted as an absence of prognostic relevance. When a downstream mediator—tumor stage—is included in the model, the indirect causal path of the upstream variable is partially absorbed, attenuating its apparent direct effect—a well-recognized phenomenon in causal mediation analysis. In addition, certain subgroups had very small sample sizes, leading to wide confidence intervals and insufficient statistical power. This interpretation is further supported by Hussain [66], who conducted the largest SEER-based laterality analysis to date, comprising 1925 patients. In univariable analysis, unilateral cases showed a 55.6% lower hazard of cause-specific death compared to bilateral cases; however, after adjusting for demographic and clinical confounders, this cause-specific survival advantage was no longer statistically significant (left-sided: HR = 0.870, *p* = 0.722; right-sided: HR = 1.008, *p* = 0.981)—a pattern directly consistent with our multivariable CPH results. Notably, the other-cause survival model remained significant after adjustment, suggesting that the residual laterality effect on non-cancer mortality may reflect the downstream burden of germline RB1 mutations rather than a direct effect on retinoblastoma-specific outcomes. Together, these findings support the interpretation that laterality’s prognostic contribution operates primarily through its influence on disease presentation and staging. Both variables must therefore be retained simultaneously: excluding tumor stage would leave the survival pathway incompletely specified, while excluding laterality would remove the upstream structural determinant that explains why staging distributions differ systematically between patient subgroups. The integrated feature-selection framework captured this upstream causal role by identifying laterality as a structural root node in the DAG and confirming its statistical relevance at the univariate and LASSO-based regularization, retaining it as a meaningful upstream predictor even in the absence of a significant direct hazard ratio in the multivariable model.

Another important factor in our models is “RX Summ”—Surg Oth Reg/Dis, which represents surgical procedures involving the removal of distant lymph nodes or other tissues beyond the primary site [46]. In the DAG (Figure 2), “RX Summ” showed a direct structural path to the survival outcome and was also structurally connected to “SEER historic stage A”, although the causal direction between these two variables could not be uniquely resolved from observational data alone. This structural position is clinically meaningful: more advanced-stage tumors are more likely to require extensive surgical intervention beyond the primary site, and such procedures themselves reflect underlying disease severity. Had this variable been included without causal scrutiny, its association with survival could easily be misattributed to the surgical procedure itself rather than recognized as a marker of disease burden—a classic confounding scenario in observational cancer registry data. The results from the CPH model (Table 2) confirmed its strong association with increased mortality risk. The literature corroborates this finding, indicating that once cancer spreads beyond the primary site, response to conventional therapy declines, leading to higher mortality rates [67]. However, the results for “Non-primary surgical procedure to distant site” and “Non-primary surgical procedure to other regional sites” were not statistically significant, suggesting that their impact on retinoblastoma survival remains inconclusive. Further studies with larger sample sizes are needed to validate these findings. We also note that several RX Summ categories showed wide confidence intervals, and one category showed a large hazard ratio, likely reflecting sparse-cell instability due to the low event rate and small subgroup counts. Therefore, the CPH coefficient estimates for RX Summ should be interpreted cautiously as predictive model parameters rather than stable causal or etiologic effect estimates.

The “sequence number” variable, which counts all reportable tumors diagnosed in a given year, was also included in our models. This factor helps identify patients with only one malignant primary tumor over their lifetime, improving the accuracy of survival analysis [46]. Retinoblastoma is a rare retinal tumor commonly associated with mutations in the RB1 oncogene, which increases the risk of secondary malignancies [68]. In the learned DAG (Figure 2), sequence number showed a direct structural path to the survival outcome as a Tier 1 predictor, independent of the other retained variables. This structural independence is important: the prognostic contribution of sequence number reflects a distinct causal pathway—most plausibly the cumulative physiological burden and systemic vulnerability associated with multiple primary malignancies—rather than acting as a proxy for tumor stage or treatment type. Without DAG-based evaluation, this variable might have been treated as statistically redundant with other tumor-related features, or included without recognizing its independent causal relevance. The results from the CPH model (Table 2) confirmed its role as a significant risk factor: patients diagnosed with their first of two or more primaries exhibited a substantially elevated hazard of death. These findings are consistent with previous studies [69,70], which suggest that long-term survival remains affected by the occurrence of secondary malignancies.

Taken together, these predictors reflect different but complementary aspects of prognosis, including tumor extent, treatment-related context, and broader disease history. The causal structure revealed by the DAG (Figure 2) further illustrates how these variables form a coherent causal network rather than parallel independent risk factors: laterality shapes staging, staging influences surgical decision-making, and the cumulative tumor burden encoded in sequence number operates through a distinct independent pathway. This layered causal architecture supports the view that capturing structural relationships—rather than selecting variables on statistical grounds alone—is essential for building both accurate and interpretable prognostic models, while also providing a biologically and clinically coherent explanation for the final predictor set.

This interpretation also helps explain why these four predictors were retained through the integrated feature-selection framework. In the univariate CPH analysis, they demonstrated prognostic relevance through their association with survival outcomes; in the PC algorithm-based causal screening, they occupied structurally meaningful positions within the learned DAG; and in the LASSO regularization step, they were also included among the LASSO-selected variables. This consistency suggests that the final predictors were not selected by any single criterion alone, but instead captured complementary dimensions of retinoblastoma prognosis, including disease presentation (laterality), tumor extent (SEER historic stage A), treatment-related disease burden (RX Summ), and long-term cancer history (sequence number). Thus, the integrated retention of these predictors helps bridge the methodological contribution of the proposed causality-informed feature selection framework with its clinical relevance for prognostic assessment.

Beyond predictive performance, the causality-informed selection strategy may improve interpretability by linking the retained predictors to a plausible data-generating structure rather than relying on empirical associations alone. However, because this study did not include an external validation cohort, improved transportability cannot be claimed from the present data. The comparison between the LASSO-only CPH model (C-index = 0.652, nine variables) and the causally refined CPH model (C-index = 0.687, four variables) illustrates this point in the present dataset: the proposed strategy reduced the feature set while achieving higher internal test-set discrimination and improving interpretability. Nevertheless, this finding should be interpreted cautiously, as whether these advantages translate into better external performance remains an important hypothesis for future validation. This is particularly relevant in rare-disease settings where the risk of overfitting and spurious feature selection is elevated due to limited sample sizes and low event rates. Furthermore, the consistency of variable retention across all six plausible DAG configurations provides evidence that the structural conclusions are robust to residual directional uncertainty in the learned graph, rather than dependent on any single assumed causal orientation.

**Figure 7:**
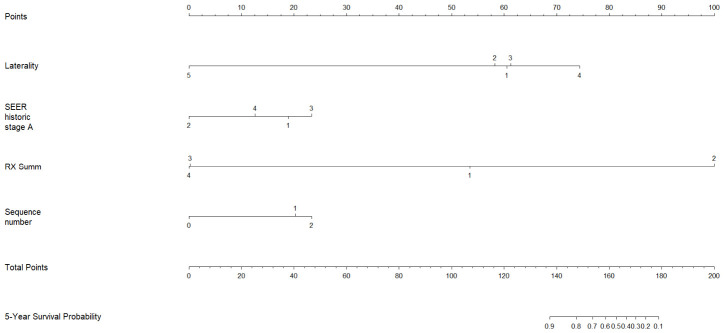
Nomogram model of 5 years OS in patients with retinoblastoma.

Although RSF achieves high accuracy and is very convenient for predicting the prognosis of retinoblastoma patients, as it allows doctors to input the patient’s overall data into the program and instantly obtain the prognosis, providing a convenient tool for prognosis assessment, there are also challenges in hospitals lacking modern equipment. Compared with conventional CPH regression, the proposed framework requires more computational and methodological support during model development, such as IPCW adjustment, causal structure learning, and ML-based survival modeling. These requirements can influence its adoption in some clinical research settings, particularly where statistical expertise, programming experience, or software support is limited. However, most of this complexity occurs during model development rather than during routine model use. Once the model has been trained and validated, the final RSF model can generate individualized predictions efficiently using routinely collected clinical variables. In hospitals with adequate computational resources, the RSF model can therefore be used directly for individualized prognostic prediction. In resource-limited settings, a classic nomogram based on the CPH model, like the one shown in Figure 7, can be a useful alternative. This nomogram was constructed based on the four most important factors identified by the CPH model, and although it achieves a C-index of 0.687 on the test set, its simple and visual nature makes it extremely easy to use. Because the nomogram was constructed from CPH coefficients, it may inherit the coefficient instability observed for some categorical predictors, particularly RX Summ. This may affect nomogram calibration, especially for patients in rare treatment-related categories. Therefore, the nomogram should be interpreted as a transparent supplementary tool for preliminary risk assessment, and its calibration requires further evaluation in larger external cohorts. Thus, this nomogram could be utilized in hospitals with limited resources for initial screening to identify high-risk patients, who can then be further evaluated using the RSF model for more precise predictions regarding their need for additional treatment. This complementary strategy enables flexible adaptation to local resource availability, balancing practicality with predictive accuracy.

From a clinical perspective, the improvement in discrimination from the refined CPH model (C-index = 0.687) to the RSF model (C-index = 0.739), although moderate, may enhance patient risk stratification by enabling more accurate identification of patients across different prognostic risk levels. Such improvement could help clinicians tailor follow-up intensity, optimize treatment planning, and provide more individualized prognostic assessment. Nevertheless, improved predictive performance does not necessarily translate directly into better clinical outcomes. Therefore, prospective studies and external validation are warranted to determine whether the observed performance gain leads to meaningful clinical benefits in routine practice.

Wang et al.’s study [31] has a similar research objective to our study. However, several important differences should be noted. That study drew on single-center hospital data collected between February 2012 and April 2019 (n = 320), whereas the present study used publicly available SEER data spanning 1975 to 2020, yielding a final cohort of 1015 patients after exclusion criteria were applied. For a rare pediatric malignancy with a low event rate, the broader temporal coverage of the SEER dataset is not a limitation but an advantage: it accumulates a larger number of outcome events and captures secular trends in treatment and survival across multiple decades, thereby providing a more robust empirical basis for model development than any single institutional cohort from a restricted time window. In addition, Wang et al. focused on a fixed-time binary classification framework with 5-year survival status as the target variable, whereas our study modeled both survival time and event occurrence within a time-to-event survival framework. In their research, the AUC of the XGBoost model in the training group was 0.951, significantly higher than the 0.837 of the CPH model (*p* = 0.001). However, in the validation group, the AUC of the XGBoost model dropped to 0.902, which was lower than that of the CPH model (AUC = 0.953). Moreover, the study did not include a test group, making it difficult to conclude which method has superior accuracy. Although Wang et al. compared XGBoost with Cox regression, they did not benchmark mainstream ML survival methods within a full survival-analysis setting. In contrast, our study employed a dedicated boosting method for survival analysis, namely GBST. While GBST performed slightly better than CPH on the test set, the difference was not substantial, and both RSF and DeepSurv outperformed GBST. Thus, for the retinoblastoma dataset, boosting methods may not be the optimal choice. Further research is needed to validate the impact of boosting methods on improving the accuracy of retinoblastoma prognosis prediction.

A more direct comparison can be made with the recent SEER-based RSF study by Zhang et al. [32], which used a survival modeling framework and reported excellent predictive performance. That study included SEER patients diagnosed between 2000 and 2019 (n = 577), with a data collection window that ends one year earlier and a cohort roughly half the size of ours. Importantly, only 17 of the 577 patients died, yielding an event rate of approximately 2.9%. Under such sparse-event conditions, model performance metrics are highly sensitive to the specific train–validation split chosen. The authors divided the cohort into training and validation sets in a 7:3 ratio using a single random split, reporting a training C-index of 0.9803 and a validation C-index of 0.9122 together with very high time-dependent AUCs. These results indicate that RSF can achieve high discrimination in RB prognosis prediction. However, those estimates were obtained from a single split under an extremely low-event setting, and therefore should be interpreted cautiously.

To further examine the reproducibility of such high RSF performance, we conducted an additional reproduction analysis following the published experimental setting and summarized the results across 10 random seeds (Table 4). Across these 10 reproduced random splits, the validation C-index ranged from 0.7896 to 0.9482, whereas the training C-index remained consistently high, ranging from 0.9418 to 0.9631. The validation 1-year, 3-year, and 5-year AUCs also varied across runs. Notably, the number of validation events in these reproduced splits ranged from only 5 to 13, indicating that the validation metrics were obtained under a very sparse-event setting. Taken together, these results suggest that RSF performance under this setting is sensitive to data partitioning, and that a single split may overstate the apparent stability of the model. Therefore, our findings support a more cautious interpretation of very high RSF performance in low-event RB data and further highlight the value of using a separate test set, repeated evaluation, and causality-informed feature selection in model development.

Compared with Zhang et al.’s study [32], the present study included a larger cohort and a greater number of outcome events, with 1015 patients and 87 events, and used separate training, validation, and test sets. Nevertheless, because the absolute number of events remained limited, the concern regarding partition-related instability was also relevant to our own model comparisons. To address this issue, we further performed a repeated random-split robustness analysis using 100 independent 8:1:1 splits. As shown in Table 5, the improvement from the LASSO-only Cox model to the causally refined Cox model observed in the original split was not consistently reproduced across repeated splits, suggesting that this apparent increase may have been partly influenced by partition-related variability. However, the causally refined four-variable Cox model achieved a test C-index comparable to that of the LASSO-selected nine-variable Cox model [0.6789 (IQR: 0.5990–0.7448) vs. 0.6894 (IQR: 0.6003–0.7647)], indicating that the four causally refined predictors retained most of the prognostic information while offering a more parsimonious and clinically interpretable feature set. More importantly, RSF maintained a clear advantage over Cox across repeated splits. Using the causally refined four-variable set, RSF achieved a higher median test C-index than Cox [0.7567 (IQR: 0.6672–0.8015) vs. 0.6789 (IQR: 0.5990–0.7448)]; using the LASSO-selected nine-variable set, RSF also outperformed Cox [0.7577 (IQR: 0.7144–0.8144) vs. 0.6894 (IQR: 0.6003–0.7647)]. These findings suggest that although discrimination estimates remain sensitive to partitioning in low-event RB data, the relative advantage of RSF over Cox was generally preserved, while the causally refined four-variable set provided a more concise and interpretable alternative with largely preserved predictive information. Detailed event counts and model performance across the 100 repeated splits are provided in Supplementary Table S2.

Lifelong surveillance is essential for mutation carriers due to their increased risk of secondary malignancies, and screening of family members is crucial for early detection [71]. The predictive model established in this study can help identify retinoblastoma patients at high risk of mortality, enabling clinicians to make more informed decisions regarding optimal prognostic treatments and interventions. This, in turn, may improve survival rates and allow healthcare resources to be allocated more effectively to those in greatest need. However, inadequate follow-up of retinoblastoma patients, particularly in low- and middle-income countries, remains a significant challenge, contributing to poorer prognoses and higher mortality rates [72]. It is important to give special attention to patients at high risk of death identified by the nomogram to avoid a loss of follow-up and provide timely intervention.

Despite the promising results obtained in this study, several limitations should be acknowledged. The data used in this study were limited to retinoblastoma cases in the United States, which has a diverse ethnicity, but may not accurately reflect geographical differences in other countries and regions. Furthermore, there may be some selection bias present, and further external validation is needed to determine the model’s generalizability and to assess the transportability of the proposed causality-informed feature-selection strategy in independent populations. This study was retrospective in nature and only included a limited number of variables in the model; therefore, future prospective studies would be required to optimize the predictive model further. Although the proposed causal feature selection framework provides a principled approach for incorporating causal structure into predictive modeling, the inferred DAG should not be interpreted as definitive biological causation, as the PC algorithm relies on assumptions such as causal sufficiency, correct conditional independence testing, and the absence of important unmeasured confounders that are difficult to fully verify in observational registry data. Finally, while survival is a critical factor, vision quality is also a factor that affects long-term quality of life, even though it is not directly related to survival. Including vision quality as a factor in the model could be considered; however, due to the limitations of the SEER database, vision-related information is currently not available. Future work should focus on external validation in independent cohorts from different institutions or countries, incorporation of additional clinically relevant variables and vision-related outcomes, and prospective evaluation of the proposed framework to further enhance its generalizability and clinical utility.

## 5. Conclusions

This study developed and validated a causality-informed ML-based survival prediction framework for retinoblastoma using SEER data. By integrating PC algorithm-based causal structure learning into an integrated feature-selection framework, four causally and prognostically grounded predictors were identified: laterality, “SEER historic stage A”, “RX Summ”, and “sequence number”. Among the models evaluated, RSF achieved the best performance (C-index = 0.739, 5-year AUC = 0.713), outperforming the refined CPH model (C-index = 0.687, 5-year AUC = 0.683), and a complementary CPH-based nomogram was developed for use in resource-limited settings. These findings demonstrate that incorporating causal structure learning into survival modeling enhances both predictive performance and interpretability, and may offer a methodological template for prognostic modeling in other rare pediatric malignancies.

## Figures and Tables

**Table 1:** Characteristics of Study Population and univariate analysis results.

Characteristics	Value	*p*-Value	Characteristics	Value	*p*-Value
**Age, yrs: mean ± SD**	1.45 ± 3.41	0.749	**Sequence number, n (%)**		
**Gender: female, n (%)**	515 (50.74)	**0.0004**	One primary only	951 (93.69)	
**Primary Site, n (%)**			1st of 2 or more primaries	58 (5.71)	**<0.0001**
Cornea, NOS	1 (0.10)		2nd of 2 or more primaries	5 (0.49)	0.2208
Retina	984 (96.95)	0.997	3rd of 3 or more primaries	1 (0.10)	**<0.0001**
Ciliary Body	1 (0.10)	1.000	**First malignant primary indicator, n (%)**		
Orbit, NOS	3 (0.30)	1.000	Yes	1009 (99.41)	
Eye, NOS	26 (2.56)	0.997	No	6 (0.59)	**0.019**
**Grade, n (%)**			**Race, n (%)**		
Moderately differentiated; Grade II	41 (4.12)		American Indian/Alaska Native	31 (15.8)	
Poorly differentiated; Grade III	85 (8.54)	0.582	Asian or Pacific Islander	27 (13.8)	0.627
Undifferentiated; anaplastic; Grade IV	58 (5.83)	0.696	Black	59 (30.1)	0.575
Well differentiated; Grade I	67 (6.73)	0.580	white	55 (28.1)	0.361
Unknown	744 (74.77)	0.602	missing	10	
missing	20		**RX Summ-Surg Oth Reg/Dis, n (%)**		
**Laterality, n (%)**			None; diagnosed at autopsy	384 (98.97)	
Bilateral, single primary	273 (26.90)		Non-primary surgical procedure performed	2 (0.52)	**0.016**
Right—origin of primary	369 (36.35)	**<0.0001**	Non-primary surgical procedure to distant site	1 (0.26)	0.994
Left—origin of primary	354 (34.88)	**0.0001**	Non-primary surgical procedure to other regional sites	1 (0.26)	0.994
Paired site, but no information concerning laterality	13 (1.28)	0.986	missing	627	
Only one	6 (0.59)	0.984	**Median household income, n (%)**		
**Diagnostic Confirmation, n (%)**			<$35,000	3 (0.42)	
Clinical diagnosis only	17 (1.67)		35,000–39,999	5 (0.69)	1.000
Direct visualization without microscopic confirmation	99 (9.75)	0.986	40,000–44,999	12 (1.66)	0.996
Positive exfoliative cytology, no positive histology	2 (0.20)	0.999	45,000–49,999	34 (4.71)	1.000
Positive microscopic confirmation, method not specified	6 (0.59)	0.999	50,000–54,999	45 (6.23)	0.996
Radiography without microscopic confirmation	59 (5.81)	0.986	55,000–59,999	79 (10.94)	0.996
Positive histology	817 (80.49)	0.986	60,000–64,999	77 (10.66)	0.996
Unknown	15 (1.48)	0.984	65,000–69,999	73 (10.11)	0.996
**Histologic Type ICD-O-3 Hist/behav, n (%)**			70,000–74,999	93 (12.88)	0.996
9510/3: Retinoblastoma, NOS	923 (90.94)		>75,000	301 (41.69)	0.996
9511/3: Retinoblastoma, differentiated	43 (4.24)	0.472	missing	293	
9512/3: Retinoblastoma, undifferentiated	48 (4.73)	0.211	**Rural-Urban Continuum Code, n (%)**		
9513/3: Retinoblastoma, diffuse	1 (0.10)	0.985	Counties in metropolitan areas ge 1 million pop	413 (58.25)	
**SEER historic stage A, n (%)**			Counties in metropolitan areas of 250,000 to 1 million pop	162 (22.85)	0.239
Distant	48 (5.11)		Counties in metropolitan areas of lt 250 thousand pop	57 (8.04)	0.773
Localized	713 (75.93)	**<0.0001**	Nonmetropolitan counties adjacent to a metropolitan area	42 (5.92)	0.333
Regional	78 (8.31)	0.311	Nonmetropolitan counties not adjacent to a metropolitan area	35 (4.94)	0.452
Unstaged	100 (10.65)	**0.012**	missing	306	
missing	76		* Survival months: mean ± SD	218.57 ± 150.01	
**cancer-directed surgery, n (%)**			* Status, n (%)		
Done	139 (13.84)		Dead	87 (8.57)	
Undone	865 (86.16)	0.466	Alive	928 (91.43)	
missing	11				

* was the dependent variable. Boldface indicates the main variable headings, and bold *p*-values indicate statistical significance (*p* < 0.05).

**Table 2:** Multivariate Cox regression analysis.

Variables	HR ^a^	95% CI	*p* Value
Laterality			
Bilateral1	1		
Right	0.820	0.575–1.170	0.273
Left	0.928	0.655–1.314	0.672
Paired site	1.191	0.313–4.531	0.798
Only one side—side unspecified	0.644	0.033–12.785	0.574
SEER historic stage A			
Distant	1		
Localized	**0.553**	0.374–0.816	**0.003**
Regional	1.659	0.973–2.828	0.063
Unstaged	0.931	0.552–1.569	0.788
RX Summ			
None; diagnosed at autopsy	1		
Non-primary surgical procedure performed	**17.934**	7.700–41.770	**<0.001**
Non-primary surgical procedure to distant site	0.740	0.003–169.528	0.914
Non-primary surgical procedure to other regional sites	0.748	0.003–175.015	0.917
Sequence number ^b^			
One primary only	1		
1st of 2 or more primaries	**2.834**	1.653–4.861	**<0.001**
2nd of 2 or more primaries	2.717	0.378–19.531	0.321

^a^ HR was Hazard Ratio. ^b^ The category ‘3rd of 3 or more primaries’ in the Sequence number variable was not included in the training set due to having only one patient, leading to the absence of this dummy variable. Bold HR and p values indicate statistically significant associations with *p* < 0.05.

**Table 3:** Hyperparameter of ML Methods.

Method	Hyperparameter
RSF	Estimators: 100
	Max Depth: 10
	Min Samples Split: 10
DeepSurv	Hidden Dim: 64, 128
	Drop Out Rate: 0.5
	Learning Rate: 0.001
	L2 Regularization Coefficient: 0.001
GBST	Learning Rate: 0.01
	Max Depth: 5
	Estimators: 300

**Table 4:** Reproduction analysis of Zhang et al. RSF model across 10 random seeds.

Run	Seed	Valid. Events	Train C-Index	Valid. C-Index	Valid. 1-yr AUC	Valid. 3-yr AUC	Valid. 5-yr AUC
1	2025	9	0.9491	0.9235	0.9499	0.9338	0.9298
2	2026	11	0.9482	0.9370	0.9437	0.9451	0.9398
3	2027	8	0.9464	0.9482	1.0028	0.9738	0.9636
4	2028	6	0.9422	0.9142	0.9452	0.9452	0.9335
5	2029	9	0.9533	0.9105	0.9454	0.9166	0.8994
6	2030	9	0.9631	0.8711	0.8227	0.8675	0.8856
7	2031	6	0.9604	0.7896	0.8636	0.8824	0.8691
8	2032	5	0.9418	0.9173	0.9980	0.9484	0.9029
9	2033	13	0.9613	0.8840	0.9015	0.9203	0.8969
10	2034	7	0.9458	0.9309	0.9959	0.9850	0.9266

Note: Results were reproduced in R (version 4.2.2) following the experimental setting of Zhang et al. Run 3 (Seed 2027) Valid. 1-yr AUC > 1.000 is likely due to numerical instability under sparse events (n = 8). Valid., Validation; AUC, area under the curve.

**Table 5:** Repeated random-split robustness analysis.

Model	N Splits	Validation C-Index, Median (IQR)	Test C-Index, Median (IQR)
Cox-LASSO 9 vars	100	0.6860 (0.5923–0.7638)	0.6894 (0.6003–0.7647)
Cox-causal 4 vars	100	0.6648 (0.6065–0.7259)	0.6789 (0.5990–0.7448)
RSF-LASSO 9 vars	100	0.7546 (0.6950–0.8199)	0.7577 (0.7144–0.8144)
RSF-causal 4 vars	100	0.7186 (0.6459–0.7833)	0.7567 (0.6672–0.8015)

Note: IQR, interquartile range.

## Data Availability

Publicly available datasets were analyzed in this study. These data are available from the Surveillance, Epidemiology, and End Results (SEER) Program of the National Cancer Institute. Access to SEER data can be obtained through the SEER website: https://seer.cancer.gov.

## Supplementary Materials

The following supporting information can be downloaded at: https://www.mdpi.com/article/10.3390/medsci14030389/s1, Figure S1: ROC Curve at 5 Year OS: CPH vs RSF; Figure S2: Sensitivity analysis of the causal DAG using an IPCW-adjusted 36-month binary survival endpoint; Table S1: Hyperparameter search spaces for AI-based models; Table S2: Event counts and test-set C-index values across 100 repeated random 8:1:1 splits; Table S3: PC-algorithm settings.
