# Supplementary material for: Causality-Guided Machine Learning for Retinoblastoma Survival Prediction: Development and Comparative Evaluation Using SEER
Source: Med Sci (Basel). 2026 Jul 14;14(3):389. doi: 10.3390/medsci14030389 (PMC13413882; doi:10.3390/medsci14030389)
Supplement: Supplementary file 1 [file medsci-14-00389-s001.zip › medsci-4419977-supplementary.pdf]

Causality-Guided Machine Learning for Retinoblastoma Survival Prediction: Development and Comparative Evaluation Using SEER

Shijie Chen and Takashi Ishida *

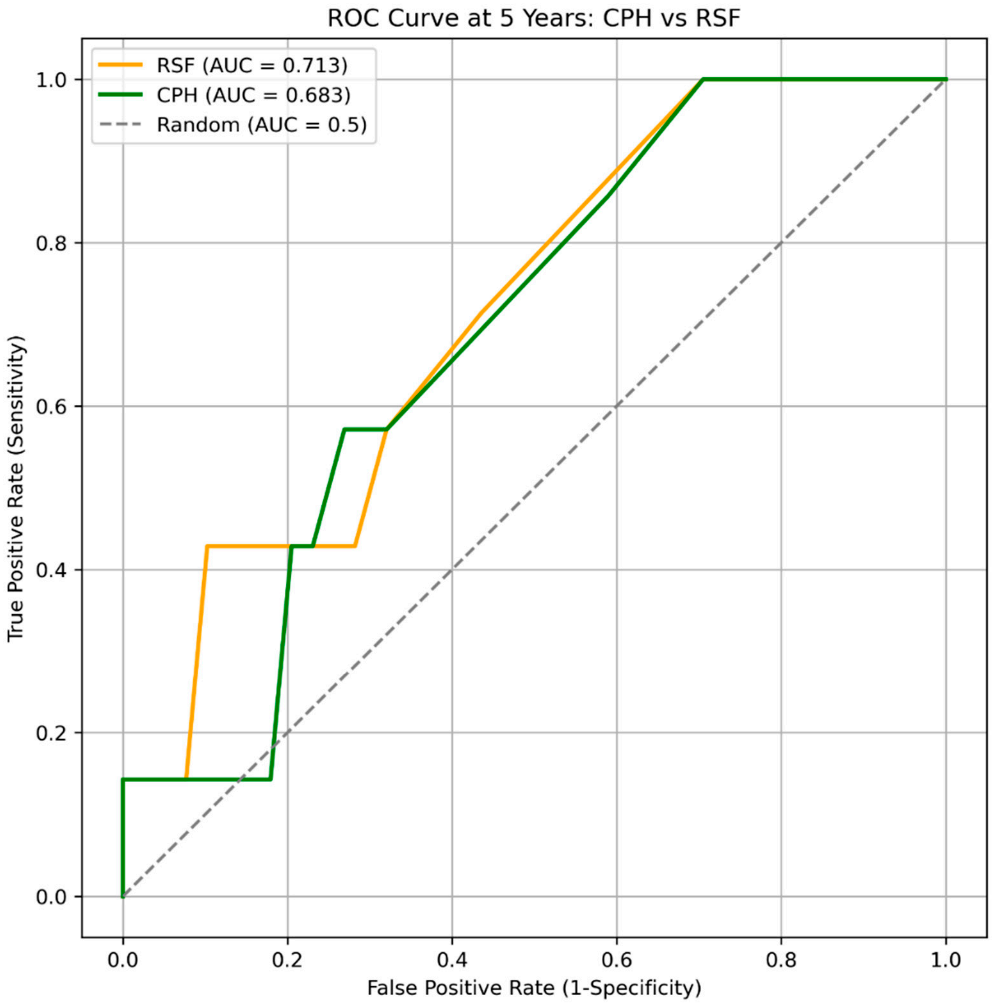

Supplementary Figure S1. ROC Curve at 5 Year OS: CPH vs RSF

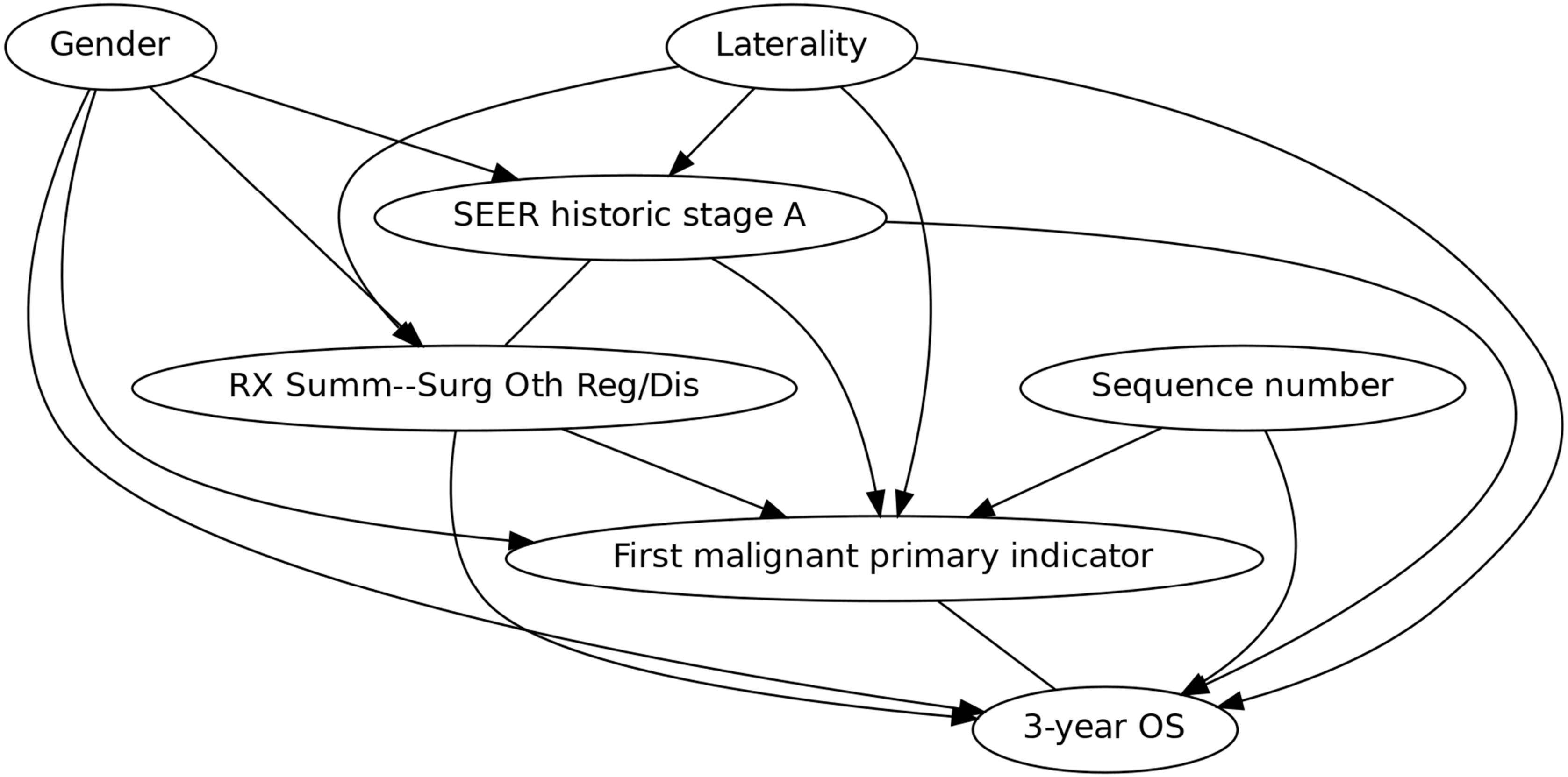

Supplementary Figure S2. Sensitivity analysis of the causal DAG using an IPCW-adjusted 36-month binary survival endpoint.

Supplementary Table S1. Hyperparameter search spaces for AI-based models

Model	Hyperparameter	Search Space
RSF	Number of Estimators	50, 100, 200
	Maximum Depth	5, 10, 15
	Minimum Samples Split	5, 10, 20
DeepSurv	Hidden Layer Dimensions	[32, 64], [64, 128], [32, 64, 32], [64, 128, 64]
	Dropout Rate	0.3, 0.5, 0.7
	Learning Rate	0.0001, 0.001, 0.01
	L2 Regularization Coefficient	0.0001, 0.001, 0.01
GBST	Learning Rate	0.01, 0.05, 0.1
	Maximum Depth	3, 5, 7
	Number of Estimators	100, 200, 300

Supplementary Table S2. Event counts and test-set C-index values across 100 repeated random 8:1:1 splits

Run	Seed	Train events	Val events	Test events	Cox LASSO test C-index	Cox causal test C-index	RSF LASSO test C-index	RSF causal test C-index
1	918273	69	10	8	0.7896	0.7300	0.7535	0.8556
2	460129	74	8	5	0.5940	0.7698	0.6294	0.7371
3	735810	78	5	4	0.6636	0.7350	0.6959	0.7627
4	192847	71	9	7	0.7708	0.6542	0.8966	0.8164
5	684205	75	5	7	0.6142	0.7301	0.7168	0.7832
6	571936	74	6	7	0.7300	0.7405	0.8143	0.7827
7	308471	73	8	6	0.6204	0.6423	0.7336	0.6442
8	846052	78	5	4	0.9470	0.9170	0.9859	0.9876
9	219583	69	7	11	0.7444	0.7938	0.8407	0.7383

10	957314	68	9	10	0.7490	0.7040	0.7040	0.5946
11	403826	77	6	4	0.6406	0.5623	0.7130	0.8000
12	682914	72	7	8	0.7146	0.8186	0.8374	0.7016
13	150739	73	5	9	0.5234	0.5879	0.6638	0.7326
14	794261	74	7	6	0.8820	0.8892	0.8737	0.9224
15	526380	67	9	11	0.7503	0.7296	0.7704	0.6068
16	871045	66	12	9	0.5933	0.4776	0.6975	0.7149
17	239416	68	11	8	0.5937	0.5379	0.8072	0.6315
18	604958	77	7	3	0.5229	0.5734	0.7064	0.7110
19	318720	62	14	11	0.6504	0.5014	0.5845	0.4585
20	965183	67	9	11	0.6098	0.6718	0.6896	0.7589
21	472609	66	12	9	0.7308	0.6037	0.8146	0.5941
22	806351	72	10	5	0.8197	0.7758	0.7918	0.6427
23	135792	69	11	7	0.7909	0.7422	0.7329	0.6014
24	690437	68	9	10	0.5044	0.4396	0.7208	0.6635
25	248615	67	11	9	0.6649	0.6693	0.8227	0.8502
26	913508	74	6	7	0.7911	0.9107	0.9125	0.9161
27	507246	71	6	10	0.7500	0.7760	0.8020	0.7229
28	761934	72	7	8	0.4932	0.4417	0.7204	0.5660
29	384120	65	11	11	0.7325	0.7431	0.8280	0.8296
30	629875	69	13	5	0.7682	0.7022	0.8598	0.8181
31	174963	66	9	12	0.5796	0.5989	0.7623	0.7477
32	852047	70	7	10	0.8094	0.7901	0.6948	0.7707
33	496318	69	11	7	0.8612	0.8603	0.8099	0.8603
34	730562	69	7	11	0.5415	0.6177	0.7571	0.6314
35	285109	79	4	4	0.7368	0.6787	0.7091	0.7618

36	941736	63	13	11	0.5474	0.4539	0.7223	0.5381
37	563820	59	16	12	0.7001	0.6594	0.7615	0.5815
38	817294	73	9	5	0.5719	0.6328	0.7875	0.8578
39	329681	75	4	8	0.7364	0.6182	0.7492	0.7316
40	705438	67	13	7	0.6635	0.5990	0.6577	0.5260
41	198506	73	5	9	0.6426	0.6809	0.6227	0.7702
42	674921	67	8	12	0.8629	0.8788	0.8849	0.9412
43	450783	73	10	4	0.9116	0.9282	0.9061	0.9613
44	926154	67	7	13	0.8398	0.8013	0.8318	0.7933
45	537092	67	12	8	0.5710	0.6104	0.7334	0.7681
46	801465	74	5	8	0.7932	0.7121	0.8289	0.8914
47	264738	62	11	14	0.5865	0.5624	0.7324	0.6590
48	719350	71	4	12	0.5734	0.6985	0.7691	0.7144
49	386591	73	8	6	0.7958	0.7539	0.6466	0.8220
50	952806	66	10	11	0.6256	0.6004	0.7057	0.5166
51	418275	63	13	11	0.6854	0.6774	0.8347	0.7193
52	603149	72	7	8	0.5429	0.5560	0.8048	0.8060
53	157824	68	7	12	0.8104	0.7603	0.7609	0.7591
54	840693	69	7	11	0.7289	0.6849	0.7771	0.7229
55	295761	69	7	11	0.7899	0.8145	0.7565	0.7768
56	971420	66	12	9	0.7644	0.6719	0.6504	0.5630
57	536184	73	7	7	0.8840	0.9170	0.8780	0.9320
58	782905	67	8	12	0.6024	0.6036	0.8262	0.7714
59	349617	67	11	9	0.7656	0.8006	0.7732	0.8062
60	615238	69	9	9	0.4940	0.4660	0.7477	0.6941
61	208946	70	7	10	0.6541	0.6242	0.7828	0.7640

62	893571	67	8	12	0.6779	0.6792	0.8390	0.7703
63	467130	65	11	11	0.6824	0.7204	0.8251	0.8182
64	724689	69	8	10	0.7263	0.7238	0.7570	0.7673
65	185302	69	9	9	0.5889	0.6418	0.7368	0.7775
66	950417	68	14	5	0.8096	0.7652	0.8959	0.7652
67	572864	73	9	5	0.7382	0.6728	0.7853	0.8377
68	836019	65	14	8	0.7216	0.5947	0.8303	0.7232
69	301758	68	10	9	0.8180	0.8100	0.7874	0.8309
70	649203	75	2	10	0.7449	0.7024	0.7602	0.8484
71	114895	68	13	6	0.6037	0.5887	0.6521	0.5150
72	779642	74	7	6	0.8471	0.7951	0.8073	0.7248
73	425316	67	9	11	0.7679	0.7252	0.8031	0.6763
74	908734	64	13	10	0.6522	0.5657	0.7403	0.6701
75	580271	68	14	5	0.5159	0.7262	0.4899	0.7378
76	857406	69	7	11	0.7513	0.7362	0.6922	0.5578
77	236970	67	10	10	0.7408	0.7259	0.7717	0.7753
78	694815	69	4	14	0.6216	0.6461	0.7196	0.7353
79	359128	69	7	11	0.6838	0.5631	0.7757	0.6317
80	742503	68	10	9	0.7939	0.6546	0.8928	0.8517
81	167439	71	10	6	0.4826	0.5573	0.6806	0.7517
82	930586	70	5	12	0.6846	0.6755	0.7384	0.7831
83	512704	64	12	11	0.7520	0.6989	0.8392	0.7711
84	868391	69	10	8	0.7091	0.6727	0.8636	0.8382
85	274650	68	12	7	0.6499	0.5853	0.7487	0.6607
86	701982	68	9	10	0.6971	0.7161	0.7467	0.7787
87	438517	69	8	10	0.5162	0.5253	0.7148	0.7509

88	984063	75	5	7	0.5214	0.4089	0.6403	0.5507
89	620149	63	14	10	0.6436	0.6798	0.7332	0.7563
90	795836	70	12	5	0.5160	0.5842	0.7910	0.7868
91	346280	65	15	7	0.4311	0.3322	0.6625	0.6113
92	913764	74	7	6	0.7802	0.8582	0.7582	0.7571
93	205871	66	12	9	0.7614	0.7665	0.7817	0.8393
94	678430	70	8	9	0.7086	0.6917	0.7426	0.6902
95	549216	71	6	10	0.6935	0.7500	0.7224	0.7915
96	827395	76	5	6	0.6217	0.6692	0.7389	0.8131
97	193608	69	8	10	0.6316	0.5594	0.8313	0.6997
98	760524	70	8	9	0.6044	0.6153	0.6876	0.6685
99	482917	68	11	8	0.5292	0.7697	0.6254	0.6104
100	651389	75	4	8	0.4561	0.5163	0.6048	0.5999

Supplementary Table S3. PC-algorithm settings

**PC algorithm**	**Setting**
Software package	causal-learn Python package
Software version	0.1.4.3
Python version	3.10.16
Conditional independence test	Kernel-based conditional independence test (KCI)
Significance threshold	$\alpha = 0.05$
Stable PC search	Enabled
